# Multi-Responsive Behaviors of Copolymers Bearing *N*-Isopropylacrylamide with or without Phenylboronic Acid in Aqueous Solution

**DOI:** 10.3390/polym10030293

**Published:** 2018-03-09

**Authors:** Jiaxing Li, Lei Yang, Xiaoguang Fan, Fei Wang, Jing Zhang, Zhanyong Wang

**Affiliations:** 1 School of Environmental and Biological Engineering, Liaoning Shihua University, Fushun 113001, China; babyli824828@yahoo.com (J.L.); feiwang1982@yahoo.com (F.W.); 66zj@163.com (J.Z.); wangzy125@gmail.com (Z.W.); 2 College of Engineering, Shenyang Agricultural University, Shenyang 110866, China

**Keywords:** poly(*N*-isopropylacrylamide), phenylboronic acid, smart responsive behavior, hydrodynamic diameter, dynamic light scattering

## Abstract

Continuing efforts to develop novel smart materials are anticipated to upgrade the quality of life of humans. Thermo-responsive poly(*N*-isopropylacrylamide) and glucose-responsive phenylboronic acid—typical representatives—are often integrated as multi-stimuli-sensitive materials, but few are available for side-by-side comparisons with their properties. In this study, both copolymers bearing *N*-isopropylacrylamide (NIPAAm), with or without 3-acrylamidophenylboronic acid (AAPBA), were synthesized by free radical polymerization, and characterized by Fourier transform infrared spectrometry, nuclear magnetic resonance hydrogen spectroscopy and gel permeation chromatography. Dynamic light scattering was used to analyze and compare the responsive behaviors of the copolymers in different aqueous solutions. Atomic force microscopy was also employed to investigate the apparent morphology changes with particle sizes. The results demonstrated that the introduction of NIPAAm endowed the composite materials with thermosensitivity, whereas the addition of AAPBA lowered the molecular weight of the copolymers, intensified the intermolecular aggregation of the nanoparticles, reduced the lower critical solution temperature (LCST) of the composites, and accordingly allowed the copolymers to respond to glucose. It was also concluded that the responding of smart copolymers to operating parameters can be activated only under special conditions, and copolymer dimension and conformation were affected by inter/intramolecular interactions.

## 1. Introduction

Poly(*N*-isopropylacrylamide) (PNIPAAm)-comprising hydrophilic amide bonds and hydrophobic isopropyl groups, empowers hydration–dehydration changes in polymer chains with ambient temperature in aqueous solutions [1]. The products with the PNIPAAm component exhibit lower critical solution temperature (LCST), and often appears as sharp decrease or increase in hydrodynamic diameter (D_H_) of polymers. Therefore, the changing trend of D_H_ along with temperature is usually employed to infer the temperature sensitivity of PNIPAAm-containing materials. Given that the overall structure and surface property of the end products can be changed by temperature control with appropriate design and simplified operation, PNIPAAm and its derivatives are widely used for drug delivery [2], biomacromolecule immobilization [3], intelligent cell culture substrate [4], artificial extracellular matrix [5] and other biomedical fields. Phenylboronic acid (PBA) can ionize in aqueous solutions to give uncharged hydrophobic forms and charged hydrophilic forms. The charged forms generate reversible covalent complexes with cis-diol compounds, thus a response to the increasing population of negatively charged PBA will result in the formation of more hydrophilic structure [6]. Glucose addition moderately changes water solubility of PBA-containing materials, which gives them attractive glucose responsiveness. This unique characteristic can be utilized to design a drug-controlled release system [7] and develop a glucose sensor [8]. It is very valuable that the elaborate combination of stimuli-responsive polymers with such advanced structure can produce novel materials with virtues from both moieties [9]. More attempts have been made to create multiresponsive materials, especially for dual responses to temperature and glucose.

Dynamic light scattering (DLS) is a technique in physics that can be applied to determine the size distribution profile of small particles in suspension or polymers in solution [10]. Because it is easy to change physical conditions of working solutions, DLS technique is often used to examine D_H_ of PNIPAAm-mediated materials changed in response to the changes of environmental parameters, in order to illustrate the stimulus responses of intelligent composites [11,12,13]. Recently, scholars had also conducted prominent works in the synthesis of smart materials carrying NIPAAm and PBA, and further investigated their dual responses to temperature and glucose by means of the DLS technique [14,15,16,17,18]. It can be seen that PBA moieties are supposed to be modified on PNIPAAm backbone, making it easier to upgrade the thermosensitivity of PNIPAAm to multiple responses to temperature, pH and glucose, so their effective applications can be further expanded. 

In our previous study, we had prepared PNIPAAm-containing copolymers and determined their temperature responses in various aqueous solutions with different temperature, pH value, species and concentrations of salts by DLS [19]. Our results demonstrated that the intervention of hydrophilic/hydrophobic reactants, salt substances and pH reagents could change the type or strength of inter/intramolecular acting forces between polymers, so the conformation changes of polymer molecules were different from what occurred in ultrapure water. If PBA is designedly introduced into the molecular segments of PNIPAAm-based copolymers, it is bound to bring inestimable impacts on the physical properties of temperature-responsive polymers, particularly for intelligent responses. The research on performance comparison between the copolymers bearing NIPAAm with or without PBA is limited, but it is the point of this study with particular emphasis on the influence of measurement temperature, pH and carbohydrate on the stimulus responses of both copolymers. Simultaneously, particle size, morphology as well as LCST are also essential to understand the interplay between smart behavior, polymer structure and driving force [20]. This is also the focus of our study.

In this study, the novel P(NIPAAm-*co*-AAPBA-*co*-HPM-*co*-TMSPM) copolymers were synthesized by free radical polymerization of *N*-isopropylacrylamide (NIPAAm), 3-acrylamidophenylboronic acid (AAPBA), hydroxypropyl methacrylate (HPM) and 3-trimethoxysilylpropyl methacrylate (TMSPM), while P(NIPAAm-*co*-HPM-*co*-TMSPM) copolymers prepared from the same synthesis conditions were used as opponents. The following measurements were performed in succession to analyze and compare the copolymers bearing NIPAAm with or without AAPBA. Fourier transform infrared (FT-IR) spectrometry, nuclear magnetic resonance hydrogen (^1^H-NMR) spectroscopy and gel permeation chromatography (GPC) were employed to identify the chemical structure, molecular composition and molecular weight of the end products. Importantly, DLS technique was applied to determine the D_H_ of both copolymers in ultrapure water and aqueous solutions with different temperature, pH value and glucose concentration, so as to analyze the influences of inter/intramolecular interactions on copolymer size and conformation. Atomic force microscopy (AFM) were used to display visually the particle sizes of the copolymers deposited on glass surfaces, which can offer more auxiliary information for smart responsive behaviors of the copolymers.

## 2. Materials and Methods

### 2.1. Materials

NIPAAm, HPM, TMSPM and 2,2-azobisisobutyronitrile (AIBN) were purchased from Aladdin (Shanghai, China). AAPBA was supplied by J&K Scientific (Beijing, China). NIPAAm, AAPBA and AIBN were fully recrystallized respectively followed by freeze-drying. The others including hydrochloric acid (HCl), sodium hydroxide (NaOH), absolute ethanol and glucose were provided by Aldrich. UHQ water was derived from Millipore-Q ultrapure water purification system (Millipore Corporation, Molsheim, France). 

### 2.2. Copolymer Synthesis and Identification

The synthesis and identification of the copolymers bearing NIPAAm with or without AAPBA were conducted according to the reported protocols with some modifications [19,21,22]. Briefly, P(NIPAAm-*co*-AAPBA-*co*-HPM-*co*-TMSPM) (PNAHT for short) copolymers and P(NIPAAm-*co*-HPM-*co*-TMSPM) (PNHT for short) copolymers were synthesized by free radical polymerization of NIPAAm, AAPBA, HPM and TMSPM with the initial molar ratios of 20:1:1:1 and 20:0:1:1 respectively, initiated by AIBN (1% of total molar quantities for all reactants) at 60 °C for 12 h under nitrogen, then precipitated and freeze-dried under vacuum for 24 h, and finally stored in a refrigerator for further use.

The FT-IR spectra were used to analyze the functional groups of the copolymers via Nicolet Magna 750 FT-IR spectrometer (Nicolet Instrument Corporation, Madison, WI, USA) in the wavenumber range of 4000~500 cm^−1^. The ^1^H-NMR spectra were applied to identify the molecular compositions of the compounds through Bruker Avance 400 MHz NMR spectrometer (Bruker Corporation, Fallanden, Switzerland). The GPC (PL-GPC-50, Varian Inc., Palo Alto, CA, USA) was employed to determine the molecular weight distribution and polymerization degree of the composites.

### 2.3. Smart Responsive Behaviors of Copolymers

PNAHT and PNHT copolymers were respectively dissolved at the final concentration of 1.0 mg·mL^−1^ in absolute ethanol, UHQ water and aqueous solutions with different pH values (1.0~12.0) and glucose concentrations (0~6.0 mg·mL^−1^), and then stirred at room temperature for 12 h. Prior to adding 1.0 mL sample into a cuvette, the copolymer solutions were filtrated and purified with Millipore filter (0.2 μm) to remove any impurities. The cuvette was then placed in the bath of Malvern DLS Nanosizer (NanoZS90, Malvern Instruments Ltd., Malvern, UK) with scattering angle fixed at 90°. The measurements were performed from 15 °C to 40 °C with lower temperature fluctuation (±0.1 °C). The pH values or glucose concentrations of the solutions to be tested were modulated by changing the content of HCl or NaOH or glucose. To improve accuracy and comparability of experimental results, the data was collected after 10 min when the solutions had reached the setting parameters. Each correlation curve was accumulated 30~50 times to eliminate noise, and at least three replicates were carried out under the same conditions. The average value ± standard deviation was applied to present the D_H_ of copolymers obtained by volume distribution under different conditions. DLS data were analyzed using Malvern Zetasizer Software v7.11. 

### 2.4. Morphology Analysis of Glass Surfaces with Copolymer Deposition

The glass coverslips were treated by constant velocity-dip coating of different aqueous solutions with the same copolymer concentration of 1.0 mg·mL^−1^, which allowed for observing the particle sizes of PNAHT and PNHT copolymers under various working conditions. The fast-drying technique was also introduced to keep the copolymer particles deposited on glass surfaces with original states as much as possible in respective solutions in spite of some conformation changes caused by dehydration. The outlet air temperatures of dryer were in accordance with the temperature of working solutions as closely as possible. Then the morphology of coated coverslips were scanned by AFM (solve P47, NT-MDT Spectrum Instruments, Moscow, Russia). Three trials were randomly selected in each sample with different scanning sizes. The surface characteristic parameters of samples were obtained from Nova 1.1.0.1918 Software.

## 3. Results and Discussion

### 3.1. Copolymer Synthesis and Identification

#### 3.1.1. Synthesis of PNAHT and PNHT Copolymers

**Figure 1:**
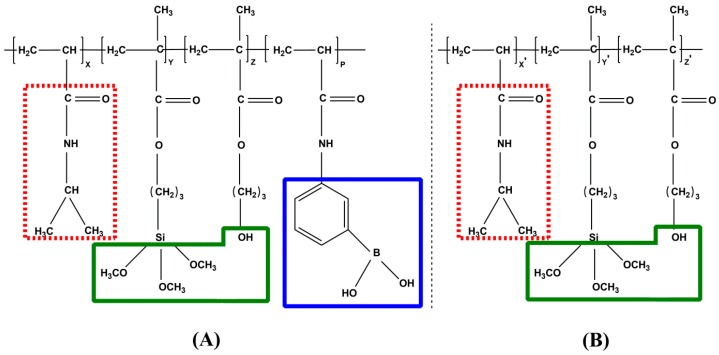
Molecular structure of PNAHT (**A**) and PNHT (**B**) copolymers.

PNAHT and PNHT copolymers have been shown to have good responses to temperature or glucose in aqueous solutions on the evidences of the follow-up measurements, but the intelligent copolymers in their current forms of molecular chains will not be used for practical applications. However, both copolymers bearing siloxane bonds can couple with hydroxyl-contained substrates with any shape and dimension by heating or radiation [23]. Meanwhile, HPM can provide effective hydroxyl groups for intermolecular bonding of copolymers, so there are nanoparticles depositing on two- or three-dimensional surfaces to form films with certain thickness. It can be concluded that synthesis of copolymers with smart responses is the premise and foundation for subsequent preparation of functional films. Furthermore, copolymer performance in response to environmental factors directly decides further applications of copolymer films. Therefore, component kinds and compounding ratios of resultant composites are key elements for film preparation, performance tuning and scheduling optimization. NIPAAm, AAPBA, HPM and TMSPM selected in this study are all unsaturated monomers with double bonds. The copolymers can be synthesized from three or four monomers by free radical polymerization, where NIPAAm and AAPBA used as primary reactants will provide their functional groups which enable them to have temperature and glucose responses. The recent studies suggested that larger PBA content gave rise to stronger glucose responsiveness for the end products [6,17,24], but excessive dosage could restrict the thermo-sensitivity of copolymers. Actually, we attempted various mole ratios of NIPAAm to AAPBA (such as 20:1, 10:1, 5:1 and so on), but it was found that adding overmuch AAPBA to the composites would result in interchain aggregation even at room temperature, and the formed clusters were difficult to pass through Millipore filters. Thus, the mole ratio between NIPAAm and AAPBA was designed to 20:1 for PNAHT copolymers. HPM and TMSPM can provide sufficient hydroxyl groups and siloxane bonds for the formation of intelligent copolymer films. It is recommended to use 5% addition of NIPAAm for both HPM and TMSPM [25]. So the initial molar ratios of NIPAAm, AAPBA, HPM and TMSPM were determined to be 20:1:1:1 and 20:0:1:1 respectively for PNAHT and PNHT copolymers in this study. The diagrams for molecular structure of both copolymers were shown in Figure 1.

#### 3.1.2. Identification of PNAHT and PNHT Copolymers

**Figure 2:**
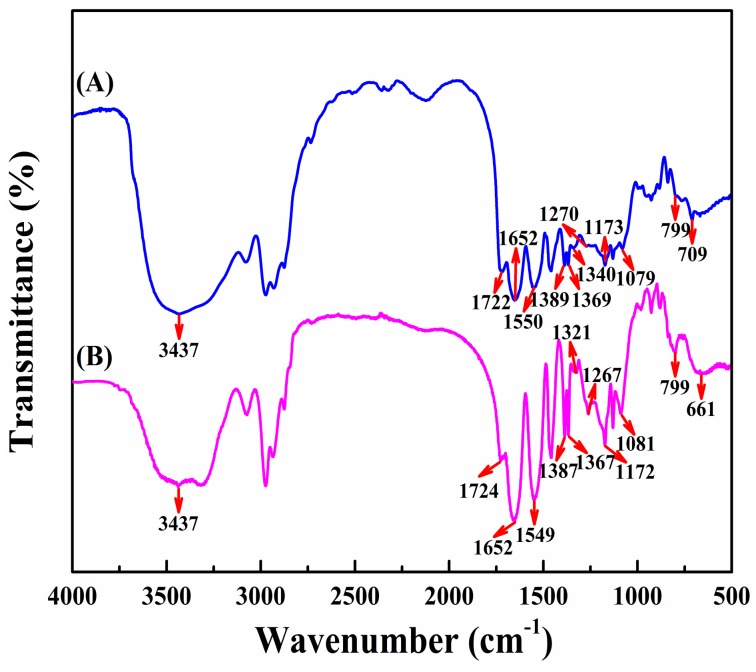
FT-IR spectra of PNAHT (**A**) and PNHT (**B**) copolymers.

Figure 2 displayed FT-IR spectra for PNAHT and PNHT copolymers. As shown in Figure 2A, the stretching vibration absorption peak of amide bond I and the bending vibration absorption peak of amide bond II were detected at 1652 cm^−1^ and 1550 cm^−1^, belonging to NIPAAm and AAPBA. The symmetrical deformation vibration absorption peaks of isopropyl bond discovered at 1389 cm^−1^ and 1369 cm^−1^, were typical absorption peaks of NIPAAm [26]. The peak at 1340 cm^−1^ was characteristic absorption peak of boric acid groups [27], and the peak at 709 cm^−1^ was the out-of-plane bending vibration absorption peak of benzene ring, which were assigned to AAPBA. The wider absorption peak appearing within 3100~3700 cm^−1^ could be due to the superposition of the stretching vibration absorption peaks of hydroxyl group and amide bond II, indirectly proving the presence of HPM, NIPAAm and AAPBA. The stretching vibration absorption peaks at 1270 cm^−1^, 1079 cm^−1^ and 799 cm^−1^ indicated that siloxane bonds were integrated into the copolymers. Absorption peaks at 1722 cm^−1^ and 1173 cm^−1^ were attributed to the stretching vibration absorption peaks of ester groups derived from HPM and TMSPM. The results confirmed that the functional groups of four reactants existed in PNAHT copolymers. The characteristic absorption peaks in Figure 2B showing a similar correlation to the results of Figure 2A except the absence of functional groups related to PBA, revealed that PNHT copolymers were synthesized from NIPAAm, HPM and TMSPM.

**Figure 3:**
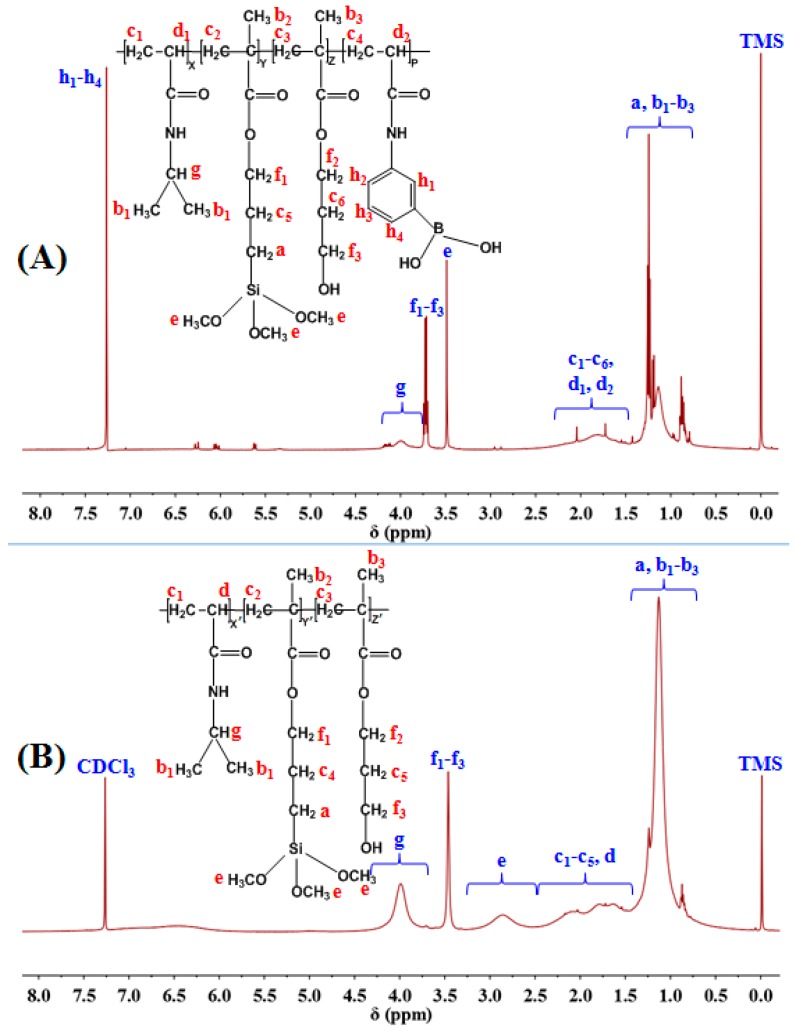
^1^H-NMR spectra of PNAHT (**A**) and PNHT (**B**) copolymers.

Figure 3 illustrated ^1^H-NMR spectra (500 MHz, CDCl_3_) of PNAHT and PNHT copolymers respectively. It was well-known that PNHT copolymers were not involved AAPBA during synthetic process. There was a peak appearing at 7.26 ppm in Figure 3B, but the peak intensity was obviously weaker than that of PNAHT group at the same chemical shift, so it was most likely a solvent peak. The integral area of solvent peak must be excluded when analyzing PNAHT group. ^1^H-NMR spectra of PNAHT copolymers was shown in Figure 3A, as described below. δ (0.75~1.50 ppm), a (Si–CH_2_–) and b (–CH_3_); δ (1.50~2.33 ppm), c (–CH_2_–) and d (–CH–); δ (3.49 ppm), e (–OCH_3_); δ (3.71 ppm), f (–OCH_2_–); δ (3.78~4.22 ppm), g (N–CH–); δ (7.26 ppm), h (–C_6_H_4_–). The ratio of integral area of characteristic peaks was approximate to 128:71:9:6:20:4, therefore the molar ratio of NIPAAm, AAPBA, HPM and TMSPM in the end product was 20:1:1:1. For PNHT copolymers, peak distributions were as followed. δ (0.74~1.47 ppm), a (Si–CH_2_–) and b (–CH_3_); δ (1.47~2.49 ppm), c (–CH_2_–) and d (–CH–); δ (2.49~3.31 ppm), e (–OCH_3_); δ (3.46 ppm), f (–OCH_2_–); δ (3.59~4.34 ppm), g (N–CH–). The ratio of integral area of characteristic peaks was approximately equal to 128:68:9:6:20, and thus the molar ratio of NIPAAm, HPM and TMSPM in PNHT copolymers was deduced as 20:1:1. Figure 3 indicated common traits as well as differences between the two spectra. Both displayed the characteristic peaks of hydrogen derived from NIPAAm, HPM and TMSPM, however, increased chemical shift of characteristic peaks and enhanced peak intensity at 7.26 ppm proved the existence of AAPBA in PNAHT copolymers. The analysis results were consistent with the principal objective of copolymer designs.

The molecular weights of PNAHT and PNHT copolymers were determined by GPC, and polymer unit of both copolymers were deduced by ^1^H-NMR spectra, and accordingly the degree of polymerization was calculated. The data were summarized in Table 1. The results indicated that the molecular weight distributions of both copolymers were relatively narrow and the molecular weights of the end products were relatively small. It was concluded that PNHT copolymers exceeded the opponents when comparing their molecular weights and polymerization degree. This was possibly because the stereo-hindrance effect of AAPBA inhibited the polymerization of unsaturated monomers to some extent. Furthermore, we also found that the molecular weights of PNAHT copolymers got smaller with the increase of AAPBA content. The observation was also a positive evidence for the above assumption.

### 3.2. Smart Responsive Behaviors of Copolymers

#### 3.2.1. Copolymers in Absolute Ethanol

Absolute ethanol was good solvent for PNAHT and PNHT copolymers, so the trend of average hydrodynamic diameter (D_AH_) of both copolymers changing with temperature in absolute ethanol was first determined. Throughout the temperature ranging from 15 to 40 °C, D_AH_ of PNAHT and PNHT copolymers showing little changes, were 36.95 ± 2.13 nm and 43.13 ± 1.54 nm respectively, indicating that the copolymers synthesized in this study were short-chain polymers. In absolute ethanol, both copolymers had unfolded conformation without detectable nanoparticle aggregation, therefore DLS data could relatively truly represent the sizes of single chains of the copolymers. Once added AAPBA, D_AH_ of PNAHT copolymers decreased, owing to the case that the stereo-hindrance effect of PBA groups terminated the synthetic process of the copolymers in advance.

#### 3.2.2. Copolymers in UHQ Water

**Figure 4:**
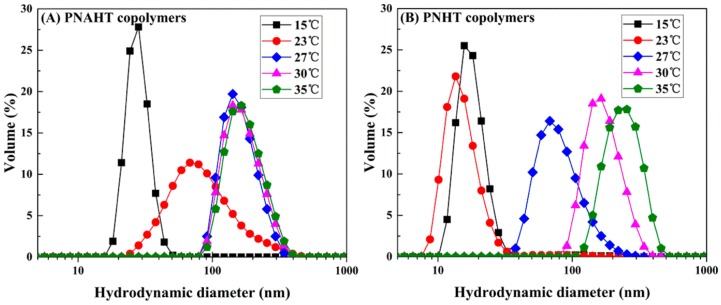
Typical D_H_ distributions of PNAHT (**A**) and PNHT (**B**) copolymers in UHQ water at different temperatures.

Figure 4 illustrated the representative DLS profiles plotted in volume distribution versus copolymer D_H_ in UHQ water. At 15 °C, D_H_ of PNAHT copolymers distributed from 15.69 to 58.77 nm with the peak appearing at 28.21 nm and PDI of 0.189, while D_H_ of PNHT copolymers distributed within 10.1~43.82 nm with the peak centered at 15.69 nm and PDI of 0.148. The results revealed that both copolymers were soluble in water without any obvious aggregation at lower temperature. But it was amazing that D_H_ of PNAHT and PNHT copolymers in UHQ water were significantly smaller than those in absolute ethanol under the same environment, indicating that the copolymers had properly adjusted their configurations of molecular chains in UHQ water. The hydrogen bonds and hydrophobic interactions produced by hydrophilic and hydrophobic groups of the copolymers in water came to a dynamic balance, so the copolymer chains actually could not extend as they did in absolute ethanol. In addition, D_H_ of PNHT copolymers were obviously lower than that of PNAHT copolymers, which would probably be the comparison between single molecular chains of PNHT copolymers and oligomers (aggregated from several molecular chains) of PNAHT copolymers. The pH of UHQ water was less than p*K*_a_ of AAPBA, therefore PNAHT copolymers were mainly uncharged and hydrophobic in UHQ water, undoubtedly increasing the hydrophobic interactions between the copolymers. However, AAPBA content accounted for only 5% of molar quantity of NIPAAm, so the hydrophilic shells were also formed by hydrogen bonds between water molecules and hydrophilic groups of the copolymers, accordingly PNAHT copolymers could only aggregate to small clusters. As a result, D_H_ of PNAHT copolymers were larger than that of control group in macroscopic manner. Obvious changes of D_H_ for PNAHT and PNHT copolymers started at 23 and 27 °C respectively, demonstrating that addition of hydrophobic AAPBA would significantly reduce LSCT of the resultant copolymers. At 30 °C, D_H_ peaks of PNAHT and PNHT copolymers shifted to 164.2 nm with distribution between 78.82 and 458.7 nm with PDI of 0.060 and 0.039 respectively, illustrating that water molecules bound with copolymers were gradually released. The continuously increased hydrophobic interactions and hydrogen bonds between the copolymers could enhance interchain aggregation, thus increasing the overall sizes of the agglomerates. As the temperature rose to 35 °C, D_H_ of PNHT copolymers continued to be expanding, with the peak at 255 nm and a wider distribution (105.7~531.2 nm) with PDI of 0.048. The volume distribution of PNHT copolymers at 40 °C was nearly the same with that at 35 °C. The results demonstrated that copolymer aggregation was constantly growing but did not unlimited, and it would be stopped once various kinds of inter/intramolecular forces kept a dynamic balance. Nevertheless, D_H_ of PNAHT copolymers had no significant changes within the temperature between 27 and 40 °C, indicating that there were only slight regulations with respect to the structure of molecular chains of PNAHT copolymers. In conclusion, when the temperature was lower than their LCST, both PNAHT and PNHT copolymers remained in hydrated state. Introduction of AAPBA obviously decreased the initial temperature for intermolecular aggregation of the constructed nanoparticles. When the temperature had risen to above LCST, the unfolded molecular chains of the copolymers began to aggregate from various directions and stopped at force balance, where large-size and widely-distributed nanometer agglomerations would be formed, showing an increase in D_H_ of copolymers.

**Figure 5:**
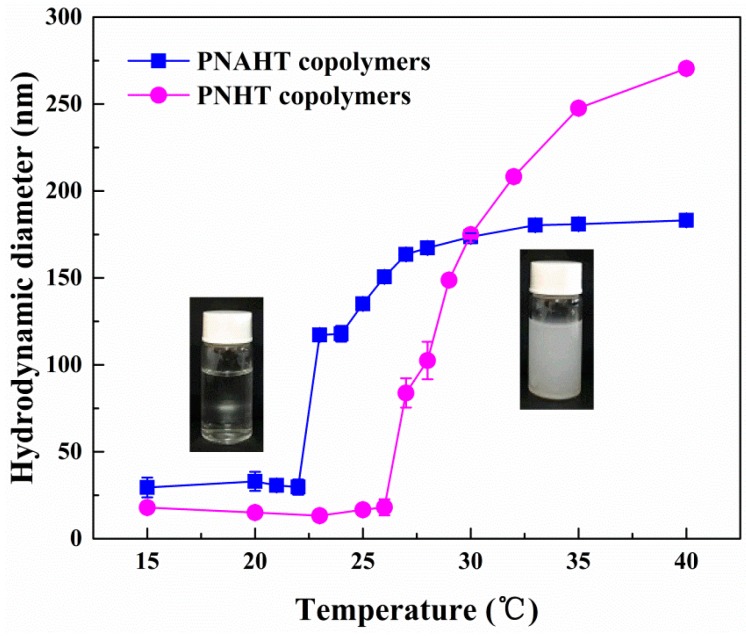
Changes in D_AH_ of PNAHT and PNHT in UHQ water as plotted with temperature.

The thermosensitive characters of the copolymers were characterized by variation tendency of D_AH_ with temperature, as displayed in Figure 5. When the temperature was lower than 23 °C, D_AH_ of PNAHT copolymers was around 30 nm, indicating that the nanoparticles were hydratable. The copolymer solution looked clear and transparent. At this point, three kinds of hydrated hydrogen bonds between water molecules and hydrophilic groups including amide bonds, hydroxyl groups and siloxane bonds, were considered as major forces to prevent intermolecular aggregation and intramolecular collapse of the copolymers. However, introduction of AAPBA would undoubtedly enhance hydrophobic interactions, leading to copolymer aggregation and LCST reduction. Across 23 °C, the scattered nanoparticles gathered obviously and become dehydrated. The transparent aqueous solution began to turn opaque. It can be deduced that the LCST of PNAHT copolymers was close to 23 °C. As the temperature continued to rise, six-fold D_AH_ could be observed, demonstrating that the molecular chains of PNAHT copolymers aggregated dramatically due to the formation of hydrophobic interactions caused by isopropyl groups and PBA groups as well as hydrogen bonds between amide bonds, but there were still three kinds of hydrated hydrogen bonds. That was to say, the maximum D_AH_ of the resultant copolymers were obtained under the actions of two kinds of hydrophobic interactions and four kinds of hydrogen bonds. Figure 5 also revealed when the temperature dropped below 27 °C, PNHT copolymers had D_AH_ of around 16 nm. The molecular chains of the copolymers started to aggregate at 27 °C, therefore the LCST of PNHT copolymers in UHQ water could be recognized as 27 °C. When the temperature was higher than LCST, D_AH_ of PNHT copolymers were elevated to 270 nm with PDI of 0.050 (40 °C), which were apparently larger than the results of PNAHT copolymers (183 nm, 0.053). This could be due to the fact that additional hydrophobic interactions derived from PBA groups led to the formation of more compact nanoaggregates.

#### 3.2.3. Copolymers in Acidic or Alkaline Solution

Pure PNIPAAm homopolymers have actually no response to pH, but the pH and temperature dual stimuli-responsive behaviors of PNIPAAm-based materials can be obtained via copolymerization with hydrophilic or hydrophobic monomers [28]. At room temperature, the solution pH of PNAHT and PNHT copolymers with final concentration of 1.0 mg·mL^−1^ were 6.2 and 6.6 respectively, showing weak acidity. Therefore, both copolymers were not easily ionized and remained uncharged forms in acidic solution, whereas they could be ionized to produce charged nanoparticles in neutral and alkaline media. Since apparent p*K*_a_ of PBA and derivatives were approximate to 8.6 [18], AAPBA addition had largely potential to delay the ionization of PNAHT copolymers.

**Figure 6:**
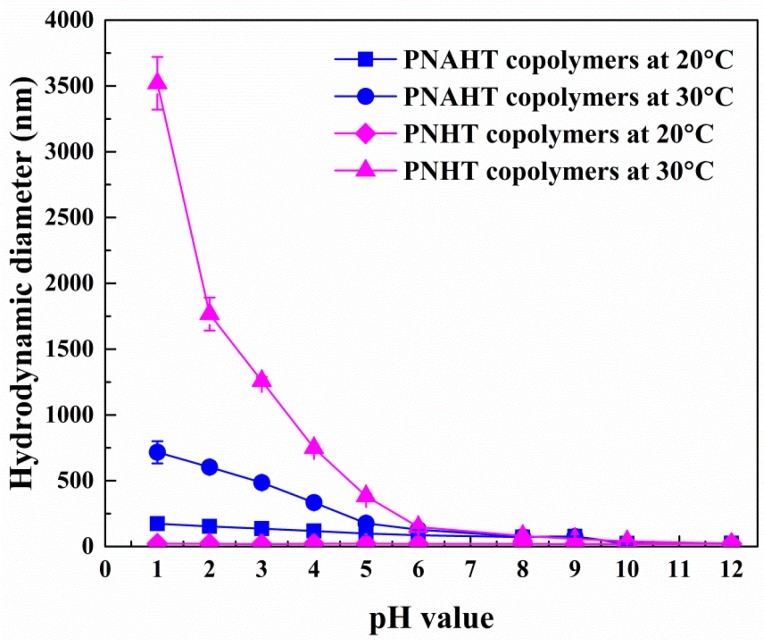
Changes in D_AH_ of PNAHT and PNHT copolymers as plotted with pH value at 20 and 30 °C.

Figure 6 showed that D_AH_ of PNAHT copolymers decreased as pH increased at 20 and 30 °C, and two variation trend curves started to be basically overlapped from pH of 8.0. When pH exceeded 9.0, D_AH_ of the nanoparticles dropped to around 19 nm, which might be due to the enhanced electrostatic repulsion and increased water solubility, proving that PNAHT copolymers were hydrated with charged single chains at this moment, thus it could be deduced that p*K*_a_ of the resultant copolymers was still within 8.0~9.0. At either 20 or 30 °C, D_AH_ of the agglomerates would be larger in more acidic environment, owing to the fact that copolymer aggregation was heightened by increasing hydrophobic interactions between PBA groups. When the ambient temperature was 30 °C and pH was lower than p*K*_a_ of AAPBA, D_AH_ of PNAHT copolymers was significantly higher than the results obtained at 20 °C with the same pH. This was mainly because the molecular chains of PNAHT copolymers were restricted by hydrated hydrogen bonds at 20 °C, D_AH_ of the agglomerates could not be infinitely expanded with the maximum value of 173 nm (PDI = 0.107). However at 30 °C, the emerging nonhydrated hydrogen bonds and hydrophobic interactions resulted in larger nanoparticles with the maximum of 716 nm (PDI = 0.088).

Figure 6 also indicated no obvious changes in D_AH_ of PNHT copolymers along with pH at 20 °C. No ascertainable aggregation or precipitation was detected throughout the entire pH range (1.0~12.0) at lower temperature. The hydrated hydrogen bonds developed by water molecules and hydrophilic groups of PNHT copolymers still played principal roles to effectively keep the dimension and structure of single chains of the copolymers. When the temperature rose to 30 °C and pH was less than 6.6, D_AH_ of PNHT copolymers would rapidly increase with the declining pH. Similarly to the situations in UHQ water, hydrophobic interactions between isopropyl groups and hydrogen bonds between amide bonds had gradually replaced the hydrated hydrogen bonds. The synergistic effect of inter/intramolecular forces triggered interchain aggregation and intrachain collapse. If in UHQ water, there were still massive hydrated hydrogen bonds, restricting further aggregation of PNHT copolymers, thus the sizes of the agglomerations could be increased only to a certain extent. However, intervention of HCl changed the action mode of solutions on copolymers [29]. By destroying hydrogen bonds between copolymers and water molecules, HCl enables hydrophobic interactions and nonhydrated hydrogen bonds to become dominant acting forces, accordingly intensifies copolymer aggregation at elevated temperatures. Higher temperature or more concentration of HCl (lower pH) gives rise to greater damage to hydrated hydrogen bonds, so larger aggregates will be observed. It was reasonably explained that D_AH_ of the agglomerations in UHQ water only reached 270 nm (PDI = 0.050) whilst D_AH_ was up to 3520 nm (PDI = 0.062) in HCl solution at higher temperature. Nevertheless, when the temperature was higher than 30 °C and pH exceeded 6.6, PNHT copolymers ionized into negative ions via deprotonation. The increasing concentration of hydroxide ions accelerated the ionization of the copolymers, leading to a large number of charged nanoparticles in aqueous solution. The effect of electrostatic repulsion reduced copolymer aggregation, so as to reduce the sizes of particle clusters.

From the above experimental results, it was clear that D_AH_ of PNAHT copolymers kept changing over pH and was independent of temperature, whereas steep declines in D_AH_ of PNHT copolymers with the increase of pH occurred only at the temperature above LCST.

#### 3.2.4. Copolymers in Glucose Solution

**Figure 7:**
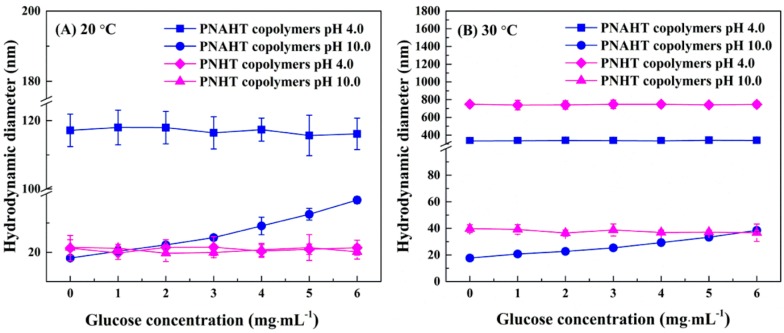
Changes in D_AH_ of PNAHT and PNHT copolymers as plotted with glucose concentration at different temperature and pH.

Considering that LCST for PNAHT and PNHT copolymers in UHQ water were around 23 and 27 °C while p*K*_a_ were within 8.0~9.0 and 6.0~7.0, the working aqueous solutions with temperatures of 20 and 30 °C, pH of 4.0 and 10.0 and glucose concentration of 0~6.0 mg·mL^−1^ were selected to investigate the glucose responsiveness of the copolymers in this study. Figure 7 displayed D_AH_ changes of both copolymers under different conditions of temperatures, pH values and glucose concentrations. It could be observed in Figure 7A, changes in pH value and glucose concentration had no significant impacts on D_AH_ of PNHT copolymers at 20 °C, and the copolymers always exhibited hydrated single chains. At 20 °C with pH of 4.0, D_AH_ of PNAHT copolymers had very low dependence of glucose concentration. PNAHT copolymers presented in the form of agglomeration at this moment, owing to the reason when pH was lower than p*K*_a_ of AAPBA, the hydrophobic uncharged PBA groups were difficult to react with cis dihydroxy groups. Therefore, glucose addition would not contribute to the rise of copolymer sizes. However, at 20 °C with pH of 10.0, D_AH_ of PNAHT copolymers increased with the increasing glucose concentration, and the maximum size was twice as much as the initial value of D_AH_ around 18 nm. As mentioned above, the combined impact of hydrophobic interactions and hydrogen bonds did not allow PNAHT copolymers to stretch themselves in UHQ water as they did in absolute ethanol, therefore copolymer sizes in UHQ water were lower than that in absolute ethanol. However, PBA groups would be converted into hydrophilic and negatively-charged phenyl borate esters in the presence of glucose. The electrostatic repulsion and hydrated hydrogen bonds enabled PNAHT copolymers with single chains to be more unfolded. Furthermore, higher glucose concentration gave rise to stronger acting forces, accordingly leading to more outstretched molecular chains of the copolymers, hence it was macroscopically reflected in the continuous expanding of copolymer sizes. The results implied that PNAHT copolymers had a glucose-stimulated response.

As shown in Figure 7B, D_AH_ of PNHT copolymers in the aqueous solution with working pH of 4.0, was significantly larger than that in the solution with pH of 10.0 at 30 °C, but addition of glucose with the increasing concentration did not cause the copolymers to respond. At 30 °C with pH of 4.0, D_AH_ of PNAHT copolymers did not show clear changes with the variation of glucose concentration, but the acidic media with the temperature higher than LCST facilitated the copolymers to gather together. At 30 °C with pH of 10.0, the variation trend for D_AH_ of PNAHT copolymers plotting with glucose concentration was consistent with the results under the condition of 20 °C at pH 10.0, which also confirmed that PNAHT copolymers had a response to glucose. For PNAHT copolymers, effective glucose response could only have occurred at the ambient pH above p*K*_a_ of AAPBA, and the ability to respond increased with the increase of glucose concentration, but it did not associate with temperature changes. The dimension and conformation of PNAHT and PNHT copolymers under different working conditions were summarized in Table 2.

#### 3.2.5. Smart Responsive Behaviors of Copolymers

PNAHT copolymers contained temperature-responsive PNIPAAm and glucose-responsive AAPBA, so they independently or simultaneously exhibited multiple responses to temperature, pH and glucose under suitable conditions. Regardless of whether the ambient temperature exceeded LCST or not in the absence of glucose, copolymer D_AH_ could shift with the changes of environmental pH, thus PNAHT copolymers had shown temperature and pH-stimuli responsiveness; only if pH of working solutions was above p*K*_a_ of AAPBA, the reversible covalent binding between charged copolymers and glucose could be formed, indicating that PNAHT copolymers were temperature-, pH- and glucose-sensitive, whereas the copolymers were hesitant to bind with glucose at relatively lower pH, which had limited capability to response. D_AH_ of PNHT copolymers fell sharply as pH grew when the temperature was higher than LCST, revealing that the copolymers had high sensitivity to temperature and pH, whereas the copolymers had failed to respond to acidic or alkaline media at lower temperature. Furthermore, thermos-responsive polymers were not dependent on glucose concentration in any case. It can be concluded that the responding of PNIPAAm and PBA to temperature, pH and glucose can be activated only under special conditions; there is no response if the activation conditions are not satisfied. Therefore, it is necessary to adjust the environmental factors to the optimal conditions to give full play their intelligent responsive performances when applied.

In summary, the major forces affecting temperature and glucose responsive behaviors of PNAHT and PNHT copolymers, include hydrated hydrogen bonds, non-hydrated hydrogen bonds, hydrophobic interactions, electrostatic interactions and so on. When the temperature is lower than LCST of the copolymers, hydrated hydrogen bonds formed by water molecules and hydrophilic groups including amide bonds, hydroxyl groups and siloxane groups, maintain the structural organization of PNAHT and PNHT copolymers with single chains or oligomers, while hydrophobic interactions between PBA groups cause the intrachain coiling of copolymer segments to slightly decrease the copolymer sizes. When the temperature is higher than the LCST of the copolymers, nonhydrated hydrogen bonds between amide bonds and hydrophobic interactions between isopropyl groups and PBA groups, trigger intermolecular aggregation and intramolecular collapse of copolymer segments, resulting in the fact that D_AH_ of the copolymers will be multiplied several times. When pH is lower than p*K*_a_ of AAPBA, HCl addition severely destroys hydrated hydrogen bonds, eventually helping non-hydrated hydrogen bonds and hydrophobic interactions become dominant driving forces, even if the temperature is lower than LCST, PNAHT copolymers will gather together to some extent. When pH is higher than p*K*_a_ of the resultant copolymers, introduction of NaOH or glucose changes the ionization equilibrium of the copolymers, correspondingly forming a large amount of charged hydrophilic nanoparticles. The emerging electrostatic repulsions and dramatically increased water solubility inhibit copolymer aggregation, accordingly cause the agglomerates to dissociate into single-stranded or oligomeric forms and make their conformations more stretched. In conclusion, the dimension and configuration of temperature or glucose responsive copolymers are the results of synergistic effects of various inter/intramolecular acting factors.

### 3.3. Morphology and Size of Copolymer Particles

DLS results showed that D_AH_ of PNAHT and PNHT copolymers varied significantly in different working solutions. For more clarity, the clean glass coverslips were dipping coated with the copolymer particles in diverse states, and then the coated surfaces were scanned by AFM. In order to eliminate experimental error, the same copolymer concentration and dipping speed were applied in coating processes. Furthermore, all matter became inert and incapable of reaction at the temperature upper limit of 37 °C in the air. The state of the copolymers deposited on the glass surfaces would be somewhat different from those in aqueous solutions, but fast drying was employed to keep their original shapes as much as possible.

**Figure 8:**
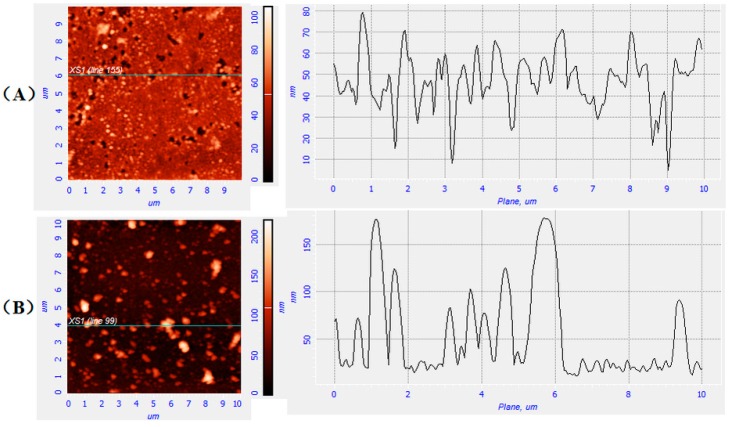
AFM images for glass surfaces with particle deposition of PNHT copolymers in UHQ water at 20 °C (**A**) and 37 °C (**B**).

Note that this project was not designed to detect the evenness of coating surfaces, so roughness made no sense. It was limited to the discussion of peak heights which reflected the typical particle sizes of the copolymer particles in this study. The height curve of X-cross section was randomly selected in each condition. The AFM images for glass surfaces with particle deposition of PNHT copolymers in UHQ water at 20 °C (Figure 8A) and 37 °C (Figure 8B) were taken as examples, as shown in Figure 8. By comparison, Figure 8A illustrated the peak heights ranging from 0 to 100 nm with the most peaks appeared at 30~50 nm, and relatively homogeneous tiny particles basically covered with the glass substrate (dark regions), whereas Figure 8B displayed the peak heights distributing from 0 to 200 nm with the peak centered at 140~160 nm, and white or almost white particles were scattered within the dark regions, which left behind greater exposed part of glass surfaces. The AFM results indicated that the particles of PNHT copolymers derived from UHQ water at 20 °C were more plentiful in number and smaller in size than those at 37 °C. The average particle sizes of PNHT copolymers at 37 °C were up to several times larger than those at 20 °C, because PNHT copolymers became reunited under the temperature above LCST but hydrated at lower temperature. The phenomenon was in agreement with DLS data. Similar findings were confirmed in other groups of PNAHT and PNHT copolymers.

## 4. Conclusions

The P(NIPAAm-*co*-AAPBA-*co*-HPM-*co*-TMSPM) and P(NIPAAm-*co*-HPM-*co*-TMSPM) copolymers were synthesized by free radical polymerization. FTIR spectra showed the characteristic functional groups of the resultant copolymers, such as amide bonds, isopropyl groups, siloxane bonds, hydroxyl groups and ester groups for both copolymers, and phenylboronic acid groups only for PNAHT copolymers. The molar ratios of NIPAAm, AAPBA, HPM and TMSPM in the final products were confirmed by ^1^H-NMR spectra to be 20:1:1:1 and 20:0:1:1 for PNAHT and PNHT copolymers. The GPC results indicated that the molecular weight distributions of both copolymers were relatively narrow, however, the molecular weight and polymerization degree of PNAHT copolymers were less than that of PNHT copolymers due to stereo-hindrance effect of AAPBA. The intelligent responsive behaviors of the copolymers in aqueous solutions with different temperature, pH value and glucose concentration were studied by DLS and AFM. The results demonstrated that PNHT copolymers were thermo- and pH-sensitive, whereas PNAHT copolymers had triple responses to temperature, pH and glucose under appropriate conditions. Furthermore, the introduction of AAPBA into PNIPAAm segments lowered the molecular weight of the resultant copolymers, intensified the interchain aggregation of the nanoparticles, and reduced the LSCT of the composites. It could be also concluded that the dimension, conformation and further LCST of the copolymers are affected by hydrous hydrogen bonds, nonhydrous hydrogen bonds, hydrophobic interactions and electrostatic interactions. Hydrated hydrogen bonds maintain the size and morphology of the nanoparticles with single chains or oligomers, nonhydrated hydrogen bonds and hydrophobic interactions lead to intermolecular agglomeration and intramolecular crimping, and electrostatic repulsions suppress intermolecular stacking and promote the extension of molecular segments.

## Figures and Tables

**Table 1:** Molecular composition of PNAHT and PNHT copolymers.

Group	Molar Ratio				*M_u_* (g·mol^−1^)	*M_n_* (g·mol^−1^)	*M_w_* (g·mol^−1^)	*M_w_*/*M_n_*	*D_P_*
	NIPAAm	TMSPM	HPM	AAPBA					
PNAHT	X	Y	Z	P	2846.76	15182	24061	1.584	6
	20	1	1	1					
PNHT	X′	Y′	Z′	P′	2655.77	19475	29115	1.495	8
	20	1	1	0					

Note: *M_u_* represents molecular weight of polymer unit; *M_n_* represents number average molecular weight; *M_w_* represents weight average molecular weight; *M_w_*/*M_n_* represents polydispersity index; *D_P_* represents degree of polymerization, *D_P_* = *M_n_*/*M_u_.*

**Table 2:** Dimension and conformation of PNAHT and PNHT copolymers under different conditions.

Item	pH	Glucose	20 °C	30 °C
PNAHT copolymers	4.0	Absence	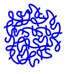	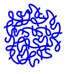
		Presence	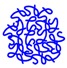	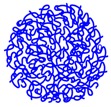
	10.0	Absence	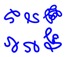	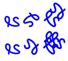
		Presence	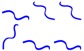	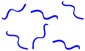
PNHT copolymers	4.0	Absence	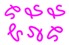	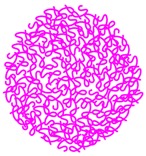
		Presence	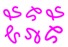	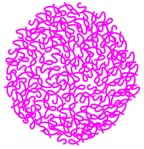
	10.0	Absence	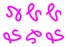	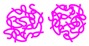
		Presence	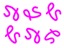	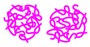
